# CARING FOR CHILDREN WITH DEVELOPMENTAL DISABILITIES: WHAT ARE THE OUTCOMES FOR HEALTH IN AGING?

**DOI:** 10.1093/geroni/igz038.1805

**Published:** 2019-11-08

**Authors:** Jessica Hoyle, James N Laditka, Sarah B Laditka

**Affiliations:** University of North Carolina at Charlotte, Charlotte, North Carolina, United States

## Abstract

Eight to ten percent of children have a developmental disability (DD). Caring for a child with DD can be rewarding. It can also be difficult, particularly for older parents or other caregivers (hereafter parents). Few studies have examined their experiences. We followed parents for 18 years using the nationally representative Panel Study of Income Dynamics (PSID). We defined DD with criteria used in research and education law, as: (1) autism, intellectual disability, learning disorder, epilepsy with seizures, attention deficit disorder/attention deficit hyperactivity disorder, cerebral palsy, or traumatic brain injury; and (2) qualification for professional services. We used three waves of the PSID’s Childhood Development Study, 1997-2007, linking children’s and parents’ PSID data (n=4,070) to study three outcomes: parents “not very” or “not at all” satisfied with “life as a whole”; nonspecific psychological distress; and reports that depressive feelings interfered with life or activities “a lot.” Discrete-time hazard models controlled for: child disability; children’s and parents’ age, sex, and education; survey design; and repeated measures. Among parents ages 47-61 at baseline (65-79 in 2015), those who cared for a child with DD were significantly more likely to report low satisfaction with life (hazard ratio, HR 4.6, 95% confidence interval, CI 1.6-13.2), distress (HR 2.8, CI 1.1-6.9) and, among women, depressive feelings (HR 1.3 CI 1.0-1.8). We found no such differences for parents less than age 65 in 2015. Results highlight the need to provide emotional support, respite, or other services for older adults caring for children with disabilities.

